# Daily, prospective associations between sleep architecture and affect: insights from Bayesian multilevel compositional data analysis

**DOI:** 10.1093/abm/kaaf050

**Published:** 2025-06-25

**Authors:** Flora Le, Yang Yap, Dorothea Dumuid, Joshua F Wiley

**Affiliations:** School of Psychological Sciences, Monash University, Clayton, VIC 3800, Australia; School of Health and Biomedical Sciences, RMIT University, Melbourne, VIC 3000, Australia; Alliance for Research in Exercise, Nutrition and Activity, Allied Health & Human Performance, University of South Australia, Adelaide, SA 5001, Australia; School of Psychological Sciences, Monash University, Clayton, VIC 3800, Australia

**Keywords:** sleep, sleep architecture, REM sleep, slow wave sleep, affect, Bayesian multilevel compositional data analysis

## Abstract

**Background:**

Emerging statistical methods addressing the multilevel compositional nature of sleep architecture can offer insights into how daily time reallocations between sleep stages (total wake time in bed [TWT], light sleep [Non rapid eye movement stage 1 and 2], slow wave sleep [SWS], and rapid eye movement [REM] sleep) are associated with post-sleep affect.

**Purpose:**

This study investigated the daily, prospective association between sleep architecture and affect.

**Methods:**

In 96 healthy, young adults across 15 consecutive days, sleep architecture was measured at night using electroencephalography (Z-Machine Insight+) and affect was self-reported using the PANAS-X at awakening. Bayesian multilevel compositional data analysis examined how reallocating time between sleep stages was associated with affect.

**Results:**

Various reallocations of sleep stages predicted affect, at both within- and between-person levels. Between-person reallocation of 30 min/night from light or REM sleep to SWS was associated with ≥0.38 points higher high and low arousal positive affect, and from SWS to any other sleep stages was associated with ≥0.21 points higher high arousal negative affect. Within-person reallocation of 30 min/night from REM to any other stages predicted ≥0.05 points higher high arousal negative affect, and 30 min/night from TWT to SWS or REM predicted ≤−0.07 lower low arousal negative affect.

**Conclusions:**

Findings highlight the distinct constellations of sleep architecture associated with affect in everyday life. Extension of SWS and REM for improving affect, while considering other off-set sleep stages, should be confirmed in experimental research in daily settings, to inform diagnostic and intervention strategies for sleep and affective disorders.

## Teaser

A novel, Bayesian multilevel compositional data analysis revealed that people with more deep sleep and rapid eye movement sleep, offset by light sleep or time awake in bed, have better emotional well-being in daily life.

## Introduction

Clinical evidence establishes the link between psychopathology and sleep, particularly the co-morbidity between insomnia and nearly all psychiatric and affective disorders,^1–3^ Conceptual models also outline the relationship between everyday sleep and affect^4–6^_ENREF_7, however, empirical evidence remains mixed, suggesting complex and nuanced relationships.^5–7^ Most evidence linking changes in sleep architecture underlying poor sleep with affect^5,8^ have been experimental in nature, with findings differing by experimental paradigms, manipulations of sleep, and measurement of affective constructs.^6^ Few studies have examined the association between sleep architecture and next-day affect, especially in naturalistic, daily life.

How time spent in bed is distributed to being awake and various sleep stages can be referred to as sleep architecture. Broadly, sleep architecture can be classified into four parts: total awake time in bed (TWT; sleep onset latency [SOL] plus wake after sleep onset [WASO]), light sleep (Non-rapid eye movement [NREM] stages 1 and 2), slow-wave sleep (SWS; also referred to as NREM stage 3), and Rapid Eye Movement (REM) sleep_ENREF_9. Experimental research on sleep deprivation and extended wakefulness periods (ie, 18 + hr)^8^ shows more TWT increases negative affect and reduces positive affect. However, studies of everyday experiences have consistently associated more TWT only with higher negative affect, not with lower positive affect.^9,10^ The associations between other sleep stages and affect are also unclear. For example, experimentally-reduced SWS (sleep continuity disruption via forced awakenings) was associated with decreased positive affect, but not negative affect.^11–13^ However, another experimental study showed that longer SWS (ie, a full night of sleep, compared to total sleep deprivation) predicted lower next-day anxiety levels.^14^ There is also conflicting evidence on whether more REM sleep results in higher,^15,16^ lower,^17^ or no change^18^ in negative affect. Most previous research is limited to the domains of experimentally induced affect and manipulated sleep (eg, deprivation or restriction). Studies should extend beyond the experimental context to examine how sleep architecture is related to affect in naturalistic, ecologically valid settings.

**Figure 1.**
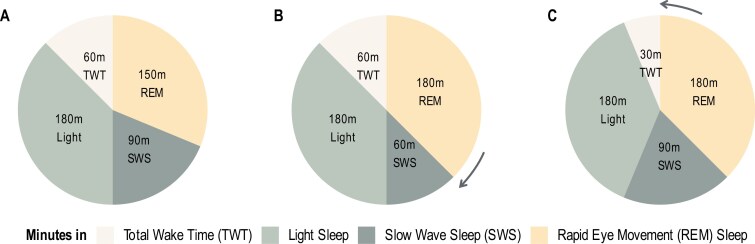
Example sleep architecture composition and hypothetical compensations. Due to the limited sleep opportunity, changes in time spent in each sleep stage can be compensated proportionally to other stages and the total time in bed. Panel A shows a hypothetical reference sleep composition. A 30-minute increase in rapid-eye-movement (REM) sleep might be at the expense of slow wave sleep (panel B), total wake time (panel C), light sleep (not shown), or a combination of these stages.

As most people have limited sleep opportunities each night, time spent in different sleep stages is often constrained within time in bed. Sleep architecture are, therefore, compositional data, whereby not only the absolute time spent in different stages, but also the relative time spent in each stage is informative. When time in bed is fixed, time spent in one sleep stage can only be increased by reducing time spent in one or more of the other stages (see Figure 1). Understanding the effect of trade-offs of time between sleep stages could clarify the existing mixed evidence regarding the role of REM sleep in facilitating emotional homeostasis^19,20^ (ie, adaptative restoration) and empirical evidence showing REM sleep deprivation did not influence affective processing.^21,22^ It may be that REM sleep is only beneficial when it is spent at the expense of specific remaining sleep stages. For example, longer REM sleep at the expense of SWS might be detrimental for affect, whereas longer REM sleep at the expense of light sleep or TWT might be beneficial. These explanations are speculative and require compositional analyses that address the effect of reallocating time between sleep stages.

Beyond the compositional nature, sleep architecture data measured repeatedly over time (eg, across days in daily studies) have a multilevel structure. Such data contain two sources of variability: between-person (differences between individuals) and within-person (deviations from an individual’s average at a given time)_ENREF_27. Limited studies have disentangled the between- and within-person effects of sleep on affect, however, those that did show that some sleep parameters (eg, sleep duration, time awake in bed, sleep efficiency) predicted affect differently at between vs within-person levels.^23,24^ No studies, to our knowledge, have explored the between- and within-person effects of daily sleep architecture composition and affect.

Furthermore, many sleep studies do not adequately distinguish affective outcomes, despite their unique associations with sleep^6^ and clinical relevance in diagnosing and treating sleep and mental health disorders.^8^ The circumplex model of affect, which posits that core affect is a blend of two dimensions of *valence* (ranging from positive to negative) and *arousal* (ranging from low to high), giving rise to four core types,^25^ aligns with the symptomology of mental health disorders. Particularly, anxiety symptoms can be described by increased high arousal negative affect (eg, nervousness and fear) and decreased low arousal positive affect (eg, reduced calmness), whereas depressive symptoms reflect elevated low arousal negative affect (eg, persistent feeling of sadness) and diminished high arousal positive affect (eg, anhedonia). Therefore, considering the distinct domains of affect in relation to sleep architecture could offer unique insights into the sleep architecture—affect relationship and the mechanisms underpinning how sleep may impact daytime emotions.

### Current study

Due to challenges in measuring and analyzing sleep architecture and affect, there is uncertainty about how different combinations of sleep architecture components are related to affect in daily life. Using at-home electroencephalographic-measured sleep architecture and EMA self-reported affect, this study:

1. Applies a new statistical method to model sleep architecture (TWT, light sleep [N1 and N2 combined], SWS, and REM), accounting for the multilevel compositional nature of sleep.
2. Examines the between- and within-person effects of sleep stage composition on affect across valence and arousal dimensions.
3. Explores whether the reallocation of time between sleep stages predicts differences in post-sleep morning affect.

## Methods

### Participants and procedures

**Figure 2.**
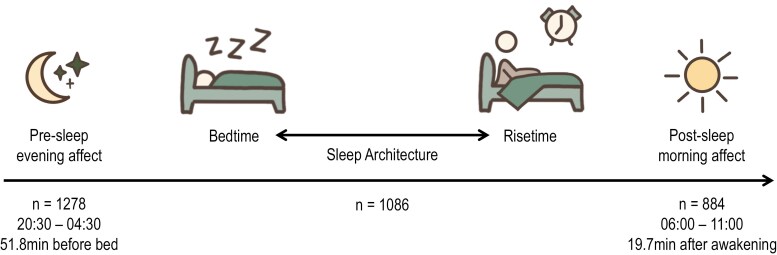
Protocol for 15-day continuous daily data collection. Sleep was recorded across the night using ZMachine. Affect was self-reported at pre- and post-sleep via MetricWire mobile phone application. Number of observations, time windows of each measure, and completion time are shown at the bottom. Of the available observations shown here, only days with complete and good quality sleep architecture (96%; all components available to construct a sleep architecture composition) and post-sleep morning affect (81%) data were used for the final analysis. The number of post-sleep affect observations was low because next-day data was not available for the first and last days of data collection.

Data was collected as part of the Stress and Health Study^26^ between February 2019 and June 2020. The recruitment process has been published previously.^26^ The study used an intensive, daily design with repeated EMA. Sleep was objectively measured using an ambulatory electroencephalographic (EEG) device (see Measures) and affect was self-reported 4 times a day (ie, awakening, afternoon, evening, and pre-bedtime) for 15 consecutive days. Two of these daily affect measures (awakening affect, pre-bedtime affect) were used in the analysis to capture post-sleep affective states (awakening affect, dependent variable) while minimizing the effect of previous-night pre-sleep affect (pre-bedtime affect, covariate) and reactivity to external daily experiences. The study procedures are in Figure 2. Overall, 96 participants completed the study and met the criteria to be included in this analysis. Monash University Human Research Ethics Committee approved this study (ID #17281), with all participants providing written informed consent. Study methods and results are reported following the Strengthening the Reporting of Observational Studies in Epidemiology Statement.

## Measures

### Sleep architecture

Sleep architecture (TWT, light, SWS, and REM sleep) was objectively measured using ZMachine Insight + (General Sleep Corporation, Cleveland, OH), a portable, single-channel EEG sleep-monitoring device. The ZMachine utilizes single-channel EEG data from two sensors placed at the mastoids to record electrical brain activity. Sleep architecture was scored using two algorithms that had a substantial agreement with technician consensus scored polysomnography^27,28^ in adults. Raw EEG signals were scored as sleep vs wake time using the Z-ALG13 algorithm with high sensitivity (95.5%) and specificity (92.5%).^27^ Sleep time was further classified into specific stages using the Z-Plus algorithm, with sensitivity ranging between 72 and 91%.^28^

### Affect

Participants reported affect at waking time using 12 affect adjectives from the Positive and Negative Affect Schedule-Expanded version scales.^29^ Four domains of affect were assessed, including high arousal positive affect (*cheerful, enthusiastic, happy*), low arousal positive affect (*at ease, calm, relaxed*), high arousal negative affect (*afraid, irritable, nervous*), and low arousal negative affect (*guilty, lonely, sad*). The items are averaged from a 5-point scale (ranging from 1-5), with a higher score indicating a higher level of affect. Between-person affect domains showed adequate to excellent internal consistency (*ω*’s ≥ 0.72). Within-person affect domains for low arousal negative affect were fair (*ω* ≥ 0.52), with other affect domains being acceptable to excellent internal consistency (*ω*’s ≥ 0.62).

### Statistical analysis

Bayesian multilevel compositional data analysis^30^_ENREF_38 was used. Compositional data analysis accounts for the relative and constrained nature of sleep architecture by quantifying the effects of increasing a specific stage while reducing one or more other stages. Specifically, we used isometric-logratio (*ilr)* coordinates that address the invalidity of using a direct composition measure (as the proportion of time spent sleeping in each stage always sums to one) and account for the interdependence of compositional parts by using a ratio. Sleep stage composition (ie, proportion of time in TWT, light sleep, SWS, and REM) was expressed as a set of *ilr* coordinates, using a sequential binary partition.^31^ Due to the multilevel nature of our data, the *ilr* coordinates were calculated at both between-person level (ie, the average time spent in each stage) and within-person level (ie, the deviation from an individual’s average time spent in each stage on a given day). Participants with complete data on sleep stages were included.

Bayesian multilevel models were fitted with the outcome being post-sleep morning affect and the predictors being between-person and within-person sleep stage composition *ilr* coordinates. All models adjusted for daily covariates, including time in bed, previous-night pre-sleep affect (to allow for a rigorous test of directionality), weekdays/weekends, circadian misalignment (measured using composite phase deviation^32^), and baseline factors, including age, gender, race/ethnicity, subjective social status, body mass index. The model was fitted with 8 chains, 8 cores, 6000 iterations including 1000 warmups (total of 40 000 post-warmup draws), and weakly informative priors. Model convergence was evaluated and confirmed using diagnostic statistic $\hat{R}$  < 1.05 and effective sample size > 400.^33^

To investigate the overall effects of between-person and within-person sleep architecture on next-morning affect, we considered four models: (1) only covariates and a random intercept, (2) model a + *between-person sleep stage composition*, (3) model a + *within-person sleep stage composition*, random slopes of the *within-person sleep stage composition* and, (4) model a + *both between- and within-person sleep stage compositions*, *random slopes of the within-person sleep architecture*.

Several methods for Bayesian model comparison using posterior distribution exist, however, recent literature suggests leaving one out (LOO)-based methods for model comparison for its robustness to models with weak priors and outliers, large datasets, and when only a few models are being compared.^34,35^ For our analysis, we employed two LOO-based methods. The first method used Bayesian stacking (of predictive distributions) which directly focuses on the performance of the combined predictive distribution to compare the weights (obtained by minimizing the LOO mean squared error) of all candidate models.^34,36^ Bayesian stacking describes the uncertainty of each model candidate using probability, with a non-zero weight indicating that adding predictor(s) improves the predictive ability of the model.^36^ The second method provides the expected log pointwise predictive density (*elpd*), an estimate of out-of-sample predictive accuracy, which can be used to compute the difference between each model’s *elpd* and the *elpd* of the best-fitting model (Δ _*elpd*_). A model with Δ _*elpd*_ = 0 is the “best” model in the set. Models with Δ _*elpd*_ > -2 were considered strong models and models with Δ _*elpd*_ < −10 were considered poor-performing.^37^ We assessed stacking weights (non-zero) and Δ _*elpd*_ (> −2) and as indicators of improved predictive performance and explored those models further in post-hoc analyses.

Post-hoc analyses, Bayesian multilevel compositional substitution,^30^ were used to explore the association between reallocating time spent between 2 specific sleep stages and affect. This analysis systematically estimates the difference in affect when increasing time spent in one stage, while decreasing another stage by the same duration, keeping the total time in bed fixed. The estimated difference in affect associated with sleep stage reallocation was calculated by the difference between affect value predicted from the mean composition and affect value predicted from a hypothetical composition where a specific time period has been reallocated between two sleep stages (eg, 30-minute more SWS and 30-minute less REM, simultaneously). This process was repeated for all possible pairwise time reallocations between sleep stages in one-minute increments from 1 to 60 minutes. Because the reverse transformation from isometric log-ratios to minutes/day results in non-linear associations, the reallocations were repeated both for adding to and deducting from, time spent in each sleep stage.

For Bayesian multilevel models, effect sizes, Bayesian (unadjusted) marginal and conditional *R*^2^ values, were reported, representing the proportion of total variance explained by fixed effects, and the proportion of total variance explained by fixed and random effects, respectively.^38^ For substitution analysis, unstandardized posterior mean differences and standardized posterior mean differences (unstandardized posterior mean differences divided by standard deviation) were computed for substitution analysis. The statistical significance of individual parameters was set as Bayesian 95% posterior credible intervals (CIs) not containing zero. As recommended by a previous review,^39^ we focused on the posterior probabilities of individual parameters, as they provide more direct answers to our research questions, while using model selection measures as supplemental.

The moderating role of time in bed in the relationship between sleep architecture and affect was also tested. The exclusion of the interaction term between time in bed and sleep architecture was supported for all types of affect, indicated by models without the interaction term that received the largest stacking weights and Δ _*elpd*_ values of 0. Analyses were performed in R^40^ and Stan^41^ (via cmdstanr), using package multilevelcoda^30,42^ (data pre-processing, model estimation, and post-hoc analyses), brms^43^ (back-end for model estimation), and loo^44^ (leave-one-out cross-validation). Analysis code is available at https://github.com/florale/multilevelcoda-sleep-stages-affect.

## Results

### Participant characteristics

Participant characteristics are in Table 1. The final sample consisted of 96 young adults (M_age_ = 20.55 years, SD_age_ = 1.65), predominantly female (77.3%), and Asian (84.5%). Participants were mostly moderate drinkers (64.9%), non-smoking (95.9%), and had an average BMI of 21.92 (SD = 3.49) within the normal range (18.5–24.9 kg/m2). On average, participants spent 449 minutes in bed per night. They spent 13% of their sleep opportunity in being awake in bed, 43% in light sleep, 21% in SWS, and the remaining 23% in REM sleep. Compared to their age group (18–34 years),^47^ participants had longer TWT (~55% [26 minutes] longer), but only a slight difference in the other sleep stages (~2% longer SWS), ~6% longer REM sleep, and ~10% shorter light sleep). High and low arousal positive affect domains were lower and high and low arousal negative affect domains were higher than those found in good sleepers with the usual amount of sleep (approximately −0.35 and + 0.26 points, respectively).^9^ The bivariate associations between each sleep architecture component and affect are visualized in the Supplementary Materials. Intraclass correlations (ICC, between-person level variance/total variance) showed that affect had higher between-person variance (ICCs: 0.55–0.67), whereas sleep architecture had higher within-person variance (ICCs: 0.29-0.40).

**Table 1:** Participant characteristics (*N* = 96)

Characteristic	M (SD)/N (%)	*n*, ICC
Age in years (range 18–25)	20.55 (1.65)	96, –
Female sex	78 (77.3%)	96, –
Body mass index	21.92 (3.49)	96, –
Subjective socioeconomic status (range 2–9)	5.49 (1.43)	96, –
Born in Australia (no)	89 (91.8%)	96, –
Ethnicity		96, –
Asian	82 (84.5%)	
White/European	9 (9.3%)	
Other	6 (6.2%)	
Smoking status (never)	93 (95.9%)	96, –
Alcohol use^a^		96, –
Abstainer	23 (23.7%)	
Moderate	63 (64.9%)	
At Risk	11 (11.3%)	
Working status (working)	21 (21.6%)	96, –
Circadian misalignment^b^	1.27 (1.03)	757, 0.40
***Daily sleep architecture (minutes)***		
Sleep period time	448.66 (95.47)	1038, 0.34
Total awake time in bed	71.77 (42.71), 0.13*	1038, 0.32
Light sleep (NREM 1 and 2 combined)	190.78 (59.80), 0.43*	1038, 0.40
Slow wave sleep	86.58 (27.64), 0.21*	1038, 0.36
REM sleep	98.35 (39.22), 0.23*	1038, 0.29
***Post-sleep morning affect (possible range 1-5)***		
High arousal positive affect	2.49 (1.09)	714, 0.67
Low arousal positive affect	2.87 (1.10)	715, 0.56
High arousal negative affect	1.37 (0.58)	714, 0.58
Low arousal negative affect	1.33 (0.61)	714, 0.55

NREM = Non rapid eye movement REM = Rapid eye movement. *n* = number of individuals for baseline variables and number of observations for daily variables. ICC = intraclass correlation coefficient. The number of post-sleep affect observations was low because next-day data was not available for the first and last days of data collection.

*Geometric mean in the composition.

^a^measured using the World Health Organization alcohol use identification test^45^_ENREF_51. Questions 9 and 10 were removed to exclude probing potentially sensitive questions regarding harms caused by participants’ alcohol use. The first three items were used to classify participants as abstainers, moderate, or at-risk based on the National Institute on Alcohol Abuse and Alcoholism recommendations^46^_ENREF_52.

^b^Measured using composite phase deviation.

### Relationship between sleep architecture composition and affect

Model comparisons showed that, for all affect outcomes, the addition of sleep architecture composition improved the predictive accuracy of the models. Stacking weights, Δ _*elpd*_ values, along with Bayesian *R*^2^ are reported in Table 2. Based on our criteria, we found evidence of improved predictive performance for: (1) *between-person* effects of sleep architecture on high arousal positive affect, (2) *between-person* effects of sleep architecture on low arousal positive affect, (3) *between-person* and *within-person* effects of sleep architecture on high arousal negative affect, and (4) *within-person* effects of sleep architecture on low arousal negative affect. These effects were further tested in the Bayesian compositional substitution analysis, by exploring how reallocations of time across stages were associated with differences in affect.

**Table 2:** Model comparisons by stacking weights and difference in expected log pointwise predictive density.

Model	Stacking weight	Δ_*elpd*_	*R* ^2^
***High arousal positive affect***			
Model a	0.89*	0.00*	0.82 [0.80, 0.84], 0.15 [0.08, 0.23]
Model b	0.11*	−0.57*	0.82 [0.80, 0.84], 0.19 [0.11, 0.29]
Model c	0.00	−5.68	0.82 [0.81, 0.85], 0.15 [0.08, 0.24]
Model d	0.00	−6.76	0.83 [0.81, 0.85], 0.20 [0.12, 0.30]
***Low arousal positive affect***			
Model a	0.78*	0.00*	0.68 [0.65, 0.71], 0.16 [0.09, 0.24]
Model b	0.22*	−0.32*	0.68 [0.65, 0.72], 0.21 [0.13, 0.31]
Model c	0.00	−4.96	0.69 [0.65, 0.72], 0.16 [0.09, 0.24]
Model d	0.00	−5.07	0.69 [0.66, 0.72], 0.22 [0.13, 0.31]
***High arousal negative affect***			
Model a	0.56*	−0.43*	0.74 [0.71, 0.77], 0.18 [0.10, 0.28]
Model b	0.00	0.00*	0.74 [0.71, 0.77], 0.23 [0.14, 0.33]
Model c	0.00	−1.58*	0.76 [0.73, 0.79], 0.19 [0.11, 0.28]
Model d	0.44*	−0.39*	0.76 [0.73, 0.79], 0.24 [0.15, 0.33]
***Low arousal negative affect***			
Model a	0.45*	−1.63*	0.73 [0.71, 0.76], 0.18 [0.09, 0.27]
Model b	0.00	−4.48	0.73 [0.71, 0.76], 0.23 [0.13, 0.33]
Model c	0.55*	0.00*	0.75 [0.72, 0.79], 0.19 [0.11, 0.28]
Model d	0.00	−2.10	0.76 [0.72, 0.79], 0.25 [0.15, 0.35]

Δ _*elpd*_  *=* difference in expected log pointwise predictive density.

* indicates that [the inclusion of] predictors improved the model predictive ability (stacking weight > 0 and Δ _*elpd*_ > −2). *R* ^2^ values included unadjusted values and 95% credible intervals of conditional *R*^2^ and unadjusted marginal *R*^2^, where conditional R^2^ considers both the fixed and random effects, and marginal *R*^2^ considers only the variance of the fixed effects, respectively. Models: (a) only covariates and a random intercept, (b) model a + between-person sleep stage composition, (c) model a + within-person sleep stage composition, and random slopes of the within-person sleep stage composition and, (d) model a + both between- and within-person sleep stage compositions, and random slopes of the within-person sleep architecture.

### Reallocation of sleep architecture and affect

**Figure 3.**
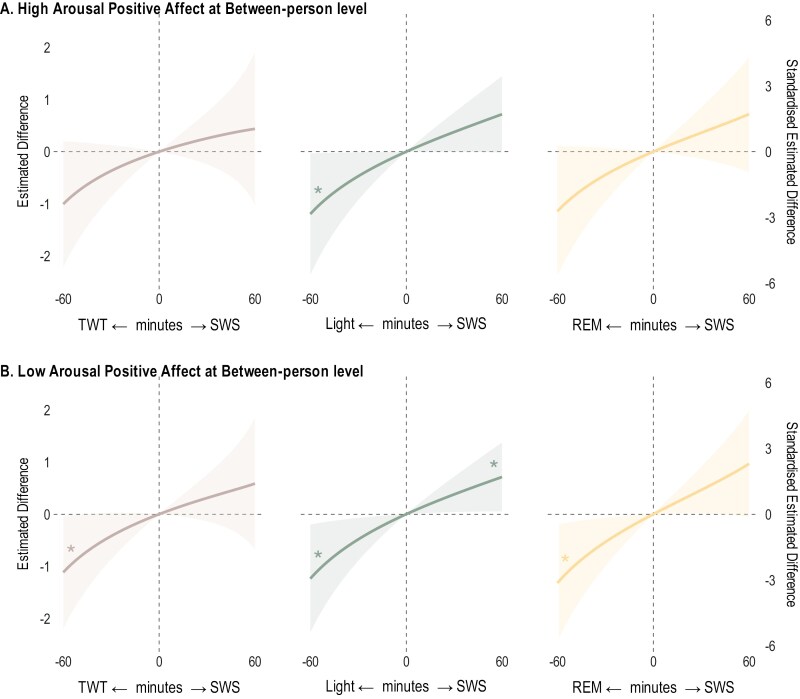
Estimated differences in post-sleep morning high and low arousal positive affect for sleep architecture reallocations from 1 to 60 minutes. All relative to the mean sleep architecture composition between-person level. TWT = total wake time in bed, light = light sleep, SWS = slow wave sleep, REM = rapid eye movement sleep. * indicates 95% credible intervals not containing 0. Models adjusted for age, sex, race/ethnicity, subjective social status, body mass index, time in bed, weekdays/weekends, circadian misalignment, and previous-night pre-sleep affect. The panels represent the pairwise reallocations. For example, the top panel shows reallocation between SWS and other sleep stages, where positive values on the x-axis (e.g., +60 minutes) indicate reallocations from another stage to SWS, whereas negative values (e.g., -60 minutes) indicate reallocations from SWS to another stage

**Figure 4.**
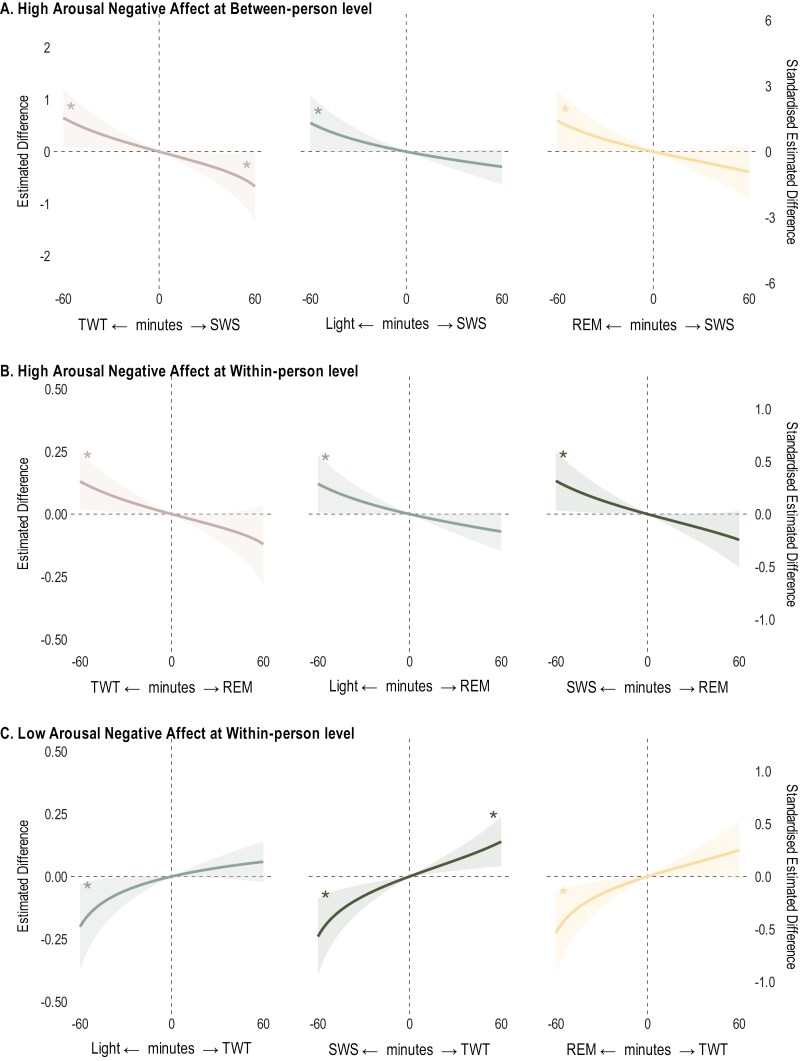
Estimated differences in post-sleep morning high and low arousal negative affect for sleep architecture reallocations from 1 to 60 minutes. All relative to the mean sleep architecture composition at within-person level. TWT = total wake time in bed, Light = light sleep, SWS = slow wave sleep, REM = rapid eye movement sleep. * Indicates 95% credible intervals not containing 0. Models adjusted for age, sex, race/ethnicity, subjective social status, body mass index, time in bed, weekdays/weekends, circadian misalignment, and previous-night pre-sleep affect. The panels represent the pairwise reallocations. For example, the top panel shows reallocation between SWS and other sleep stages, where positive values on the x-axis (e.g., +60 minutes) indicate reallocations from another stage to SWS, whereas negative values (e.g., -60 minutes) indicate reallocations from SWS to another stage.

Estimated differences (unstandardized and standardized estimates, alongside 95% CIs) in affect for 30-minute reallocations of sleep stages are in Table 3 for positive affect, and Table 4 for negative affect. For brevity, we visualized the significant results of the estimated differences (both unstandardized and standardized) in affect for reallocations of 1 to 60 minutes between sleep stages in Figures 3 and 4. We reported the unstandardized results below, with full results provided in Supplementary materials.

**Table 3:** Expected differences in post-sleep morning positive affect for 30-minute sleep architecture reallocations.

	↓ TWT	↓ Light	↓ SWS	↓ REM
***Between-person*** *sleep architecture and* ***high*** *arousal positive affect*				
↑ TWT	–	0.12, 0.15 [-0.22, 0.46]	-0.37, -0.46 [-0.86, 0.13]	0.10, 0.13 [-0.24, 0.44]
↑ Light	−0.11, −0.13 [−0.54, 0.33]	–	**−0.47, −0.59** **[−0.93, −0.01]**	0.00, 0.00 [−0.44, 0.44]
↑ SWS	0.26, 0.32 [−0.24, 0.76]	**0.38, 0.47** **[0.00, 0.75]**	–	0.36, 0.46 [−0.13, 0.85]
↑ REM	−0.08, −0.10 [−0.45, 0.29]	0.04, 0.05 [−0.34, 0.43]	−0.44, −0.56 [−0.95, 0.07]	–
***Between− person*** *sleep architecture and* ***low*** *arousal positive affect*				
↑ TWT	–	0.08, 0.10 [−0.24, 0.39]	−0.42, −0.54 [−0.87, 0.03]	0.16, 0.21 [−0.14, 0.47]
↑ Light	−0.06, −0.07 [−0.44, 0.34]	–	**−0.48, −0.63** **[−0.89, −0.07]**	0.10, 0.13 [−0.31, 0.50]
↑ SWS	0.31, 0.40 [−0.14, 0.76]	**0.38, 0.49** **[0.04, 0.72]**	–	**0.46, 0.60** **[0.02, 0.91]**
↑ REM	−0.11, −0.14 [−0.43, 0.21]	−0.04, −0.05 [−0.39, 0.31]	**−0.53, −0.69** **[−1.00, −0.07]**	–

TWT = total wake time in bed, Light = light sleep, SWS = slow wave sleep, REM = rapid eye movement sleep. Results are unstandardized mean difference, the standardized mean difference [unstandardized 95% credible intervals] for 30-minute reallocation from the behavior in the column headers to the behavior in the row headers. Bold values indicate 95% credible intervals not containing 0. Models adjusted for age, sex, race/ethnicity, subjective social status, body mass index, time in bed, weekdays/weekend, circadian misalignment, and previous-night pre-sleep affect.

**Table 4:** Expected differences in post-sleep morning negative affect for 30-minute sleep architecture reallocations.

	↓ TWT	↓ Light	↓ SWS	↓ REM
***Between-person*** *sleep architecture and* ***high*** *arousal negative affect*				
↑ TWT	–	0.05, 0.11 [−0.10, 0.21]	**0.27, 0.52** **[0.04, 0.50]**	0.02, 0.04 [−0.14, 0.18]
↑ Light	−0.10, −0.20 [−0.29, 0.09]	–	**0.21, 0.41** **[0.00, 0.42]**	−0.04, −0.07 [−0.24, 0.17]
↑ SWS	**−0.26, −0.49** **[−0.49, −0.03]**	−0.16, −0.31 [−0.33, 0.02]	–	−0.19, −0.37 [−0.42, 0.04]
↑ REM	−0.08, −0.15 [−0.24, 0.09]	0.02, 0.04 [−0.16, 0.20]	0.23, 0.45 [−0.01, 0.48]	–
***Within−person*** *sleep architecture and* ***high*** *arousal negative affect*				
↑ TWT	–	0.00, 0.01 [−0.03, 0.04]	−0.01, −0.02 [−0.05, 0.03]	**0.05, 0.13** **[0.01, 0.10]**
↑ Light	−0.01, −0.03 [−0.06, 0.04]	–	−0.01, −0.03 [−0.05, 0.03]	**0.05, 0.12** **[0.00, 0.10]**
↑ SWS	−0.01, −0.01 [−0.05, 0.04]	0.01, 0.01 [−0.03, 0.04]	–	**0.06, 0.14** **[0.00, 0.11]**
↑ REM	−0.05, −0.12 [−0.10, 0.00]	−0.04, −0.09 [−0.08, 0.00]	−0.05, −0.12 [−0.10, 0.00]	–
***Within−person*** *sleep architecture and* ***low*** *arousal negative affect*				
↑ TWT	–	0.03, 0.08 [−0.01, 0.08]	**0.06, 0.16** **[0.02, 0.11]**	**0.05, 0.13** **[0.00, 0.10]**
↑ Light	**−0.06, −0.14** **[−0.11, −0.00]**	–	0.03, 0.07 [−0.01, 0.07]	0.02, 0.04 [−0.03, 0.07]
↑ SWS	**−0.08, −0.19** **[−0.13, −0.03]**	−0.02, −0.05 [−0.06, 0.01]	–	−0.00, −0.01 [−0.06, 0.05]
↑ REM	**−0.07, −0.17** **[−0.12, −0.02]**	−0.01, −0.03 [−0.06, 0.03]	0.02, 0.04 [-0.03, 0.07]	–

TWT = total wake time in bed, Light = light sleep, SWS = slow wave sleep, REM = rapid eye movement sleep. Results are unstandardized mean difference, the standardized mean difference [unstandardized 95% credible intervals] for 30-minute reallocation from the behavior in the column headers to the behavior in the row headers. Bold values indicate 95% credible intervals not containing 0. Models adjusted for age, sex, race/ethnicity, subjective social status, body mass index, time in bed, weekdays/weekend, circadian misalignment, and previous-night pre-sleep affect.

### Sleep architecture and positive affect

For both high and low arousal positive affect, we examined the between-person effects of sleep stages and found that reallocation of SWS was associated with differences in both types of positive affect (Figure 3). Individuals who had 30-minutes longer SWS at the expense of light sleep reported higher both high and low arousal positive affect (+ 0.38 [95% CI 0.00, 0.75] and + 0.38 [0.04, 0.72], respectively). Longer SWS sleep (+ 30 minutes) at the expense of REM sleep was also associated with higher low arousal positive affect (+ 0.46 [0.02, 0.92]), but not high arousal positive affect (+0.36 [−0.13, 0.85]). Other reallocations of sleep stages were not associated with differences in either type of positive affect.

### Sleep architecture and negative affect

For high arousal negative affect, we examined both the between and within-person effects of sleep stages and found that reallocation involving all sleep parameters predicted differences in high arousal negative affect (Figure 4, panels A and B). SWS was more associated with high arousal negative affect at the *between-person* level, whereas REM was more associated with high arousal negative affect at the *within-person* level. At the *between-person* level, 30-minute shorter SWS was associated with higher high arousal negative affect (when compensated by light sleep: + 0.21 [0.00, 0.42], TWT: + 0.27 [0.04, 0.50]). The opposite direction was only supported for the TWT → SWS reallocation, with shorter TWT (-30 minutes) compensated by SWS associated with lower high arousal negative affect (−0.26 [−0.49, −0.03]). At the *within-person* level, shorter REM, regardless of which other sleep stage gained, predicted higher high arousal negative affect. On days individuals had 30-minute shorter-than-usual REM sleep, they reported higher high arousal negative affect, specifically, +0.05 [0.00, 0.10] when compensated by shorter-than-usual light sleep, +0.05 [0.01, 0.10] when compensated by TWT, and +0.06 [0.00, 0.11] when compensated by SWS.

Lastly, for low arousal negative affect, we examined the within-person effects of sleep stages and found that reallocation of TWT predicted differences in low arousal negative affect (Figure 4, panel C). Longer-than-usual TWT (+30 minutes) at the expense of SWS or REM predicted higher low arousal negative affect (+0.06 [0.02, 0.11] and +0.05 [0.00, 0.10], respectively). Longer-than-usual TWT (+30 minutes) compensated by shorter-than-usual light sleep did not predict a significant change in low arousal negative affect (+ 0.03 [−0.01, 0.08]). The opposite direction was, however, supported for all sleep stages. Other reallocations were not associated with low arousal negative affect.

## Discussion

The present study applied a novel method to examine the association between reallocation of time between sleep stages and affect, by accounting for the compositional and multilevel nature of sleep architecture. The disaggregation of the within-person from between-person effects further offers new insights into the daily relationship of sleep architecture with affect. We found sleep stages were differently associated with negative affect and positive affect at both high and low arousal levels, respectively. The effects emerged at both the within-person and between-person levels and depended on the types of affect as well as which sleep stages were involved in the reallocations.

### High and low arousal positive affect

Sleep stage composition overall was associated with positive affect (both high and low arousal) at the between-person level. Substitution analysis further revealed that reallocations of time toward SWS from other sleep stages were associated with higher positive affect. Longer SWS at the expense of REM sleep was associated with higher low arousal positive affect, whereas longer SWS at the expense of light sleep was associated with higher both high and low arousal positive affect. The association between SWS → TWT reallocation and lower low arousal positive affect was only statistically significant for reallocations of 50 minutes or more, suggesting that perhaps extended wakefulness periods, rather than normal variation in TWT, are related to reduction in positive affect.

Our work extends previous experimental findings on the positive association between SWS and positive affect,^11–13^ by showing that these associations (1) applied to both high and low arousal levels of positive affect, and (2) occurred when time spent SWS was substituted by light sleep. Notably, the finding that longer SWS at the expense of REM sleep was associated with higher low but not high arousal positive affect, may be explained by differences in mechanisms of emotional processing during these sleep stages.^4,19,20^ As REM is more sensitive to high-arousing stimuli, whereas SWS is more involved in processing neutral (ie, low-arousing) stimuli,^48^ reallocation from REM to SWS might be associated with the dissipation of emotional arousal and consolidation of neutrality, such that more resources were allocated to processing low arousal of positive affect (calmness), instead of high arousal positive affect (enthusiasm). Nonetheless, this complex mechanism requires further investigation.

### High arousal negative affect

Composition of sleep architecture was associated with high arousal negative affect at both between-person and within-person levels. At the between-person level, the sleep stage most consistently associated with high arousal negative affect was SWS. Importantly, this association was non-linear and asymmetrical, giving stronger evidence for the impact of losing SWS than extending SWS. Shorter SWS, regardless of which other sleep stage was consequently gained, was associated with higher high arousal negative affect on average. Conversely, longer SWS was only associated with reduced high arousal negative affect when SWS was increased at the expense of TWT, rather than any sleep stage. Our findings support the positive association between TWT and high arousal negative affect^10^ and the anxiolytic benefit of SWS,^14^ but differ from past studies showing a null association between SWS and negative affect.^11,12,18^ As anxiety-related symptoms can be characterized by high arousal negative affect (nervousness and fear), the null findings may be due to failure to distinguish arousal levels, whereas our data showed separatable roles of SWS in the experience of high vs low arousal negative affect.

At the within-person level, the stage most associated with high arousal negative affect was REM sleep. Reallocation of time away from REM (ie, shorter-than-usual REM) to any other stage predicted higher high arousal negative affect. A non-linear, asymmetrical association between REM and high arousal negative affect was also observed, showing the negative consequence of affective experience of losing REM sleep, but not necessarily the benefit of having more REM sleep than usual on a given day. There have been conflicting findings on whether longer or shorter REM is beneficial for emotional processing. Although REM is important for negative affect experience^17–20^ and emotional processing more broadly,^3,19–22^ longer REM (in combination with shorter REM latency resulting in increased REM pressure) has been observed in individuals with affective disorders.^1,2^ Our data suggest that REM sleep maintenance (at the individual’s habitual level), rather than enhancement or deprivation, might be most beneficial for affective experiences.

The independent roles of SWS and REM, specifically the between-person effect of SWS and within-person effect of REM sleep, align with the notion that SWS operates in the domain of a stable mood state,^14^ whereas REM is involved in the short-term, acute experience.^22,23^ Albeit weak, given the effect at a daily level, the impact of REM sleep on high arousal negative affect might accumulate when REM is continuously lost over time. Future intervention research may compare the effectiveness of interventions for acute affective symptom relief by addressing REM and those with greater treatment length aiming at extending SWS at the expense of TWT.

Interestingly, reduced REM was shown to be beneficial for (low arousal) positive affect but detrimental for (high arousal) negative affect. Thus, an optimal approach may be maintaining REM sleep at the individual’s average level, rather than increasing or reducing. Further, the detrimental effect only occurring at the within-person level highlights the role of intraindividual variability in night-to-night REM sleep in affective functioning. REM sleep regularity (eg, inconsistent night-to-night REM sleep) might be a stronger predictor of negative affect than REM sleep duration (eg, short REM sleep on average). Such intraindividual variability in sleep architecture and its relationship with affect may be explored in future research.

### Low arousal negative affect

Sleep stage composition predicted low arousal negative affect only at the within-person level. Shorter TWT, regardless of which other sleep stage was consequently gained, predicted lower low arousal negative affect. Although it is inconsistent with our previous research^10^ which found a null association between TWT and low arousal negative affect, this may be because our previous analysis explored the reallocation between TWT and total sleep duration (ie, all stages combined), rather than individual stages, and did not investigate the within-person effect. Here, we showed the affective benefit (reduced low arousal negative affect) of shortened time in TWT was non-linear, with the magnitudes depending on which of the other sleep stages were increased accordingly. This finding strengthens evidence that periods of staying awake in bed adversely influence affect^8^ and helps elucidate the interpretation of past findings regarding sleep deprivation and restriction,^4^ such that their effects on affect may be due a combination of extended wakefulness and sleep loss, rather than the absence of sleep alone.

### Strengths and Limitations

This study has several strengths. Our Bayesian multilevel compositional data analysis approach to modelling sleep architecture provided novel insights into the sleep architecture and affect relationship, specifically the differences in affect associated with reallocating time from one sleep stage to another. We robustly tested of the temporal order of this relationship by separating within-person and between-person effects, while controlling for lagged outcomes in all models. The rigorous study design, including at-home EEG-measured sleep and repeated EMAs of affect across 15 consecutive days, reduced recall biases compared to self-reported affect and addressed limitations of under- and over-estimations of accelerometer-measured sleep.

Limitations of this study include the use of a relatively healthy, young adult sample, which limits generalisability. Variation in day-to-day sleep data may be inadequate in healthy individuals, compared to clinical populations, to quantify how much sleep stage disruption is needed to alter affect. The low level of negative affect might have contributed to the null findings, although this floor effect is commonly observed in daily studies and healthy individuals.^9^ Findings require replication in clinical populations, such as those with sleep or affective disorders. Our EEG-derived sleep data were valuable in capturing sleep architecture outside the context of an in-laboratory experiment, in an ecologically valid context of the daily, naturalistic setting. Nevertheless, their specificity and sensitivity are lower than polysomnography (between 72 and 91% for sleep stage classification). Further development in at-home EEG measurements enabling daily research using EEG-derived sleep data, could improve the quality of evidence. There was an unbalanced design with more pre-sleep affect data than sleep and post-sleep affect data; however, complete data were used as methods for imputing multilevel compositional data are not yet well developed. We did not adjust for multiple comparisons due to the current lack of Bayesian methods; it is possible that some of the intervals might include zero if stringent intervals were used. Further, despite the test of directionality through time-lagged predictions and inclusion of covariates, our findings are potentially influenced by unexplored confounders. Confirmatory experimental research on the daily sleep-affect associations in naturalistic settings, using at-home sleep restriction protocols, or sleep intervention in clinical populations, could better establish causality. Given the night-to-night dependence of sleep architecture (eg, REM rebound),^49^ future research could also consider their potential impacts on the association between sleep architecture composition and subsequent affect.

### Implications and conclusions

Our study provided evidence for the relationship between sleep and affect when sleep continuity is fixed and certain sleep stages are reallocated at the expense of each other, extending the body of experimental studies that primarily focused on when sleep continuity is disrupted. The development and validation of a protocol that selectively deprives one sleep stage at the expense of another, without necessarily reducing sleep opportunity, represents a valuable avenue for experimental and longitudinal research. Experimental protocols involving SWS deprivation (eg, auditory stimulation,^50^ forced awakening during SWS^15^) or REM suppression (eg, via forced awakening during REM^15^) may use multilevel compositional data analysis to better probe the causal pathway of sleep architecture and affect. At-home auditory stimulation for SWS enhancement^51^ could be tested in relation to affect in outside-of-the-lab, longitudinal studies.

Confirmatory evidence from further research showing how combinations of sleep alterations can define distinct affective experience better than alterations in one single sleep aspect could inform designs of new evidence-based diagnostic and intervention guidelines. More optimisation of sensor technology for precise sleep architecture assessments (eg, wearable accelerometers and at-home polysomnography) that subsequently allow for accurate affect estimation in daily life could facilitate management of both sleep and affective symptoms. Assessing and tracking both affective experience and sleep architecture, in patients with sleep and affective disorders, may provide valuable insights into both short-term treatment response and long-term prognosis.

Overall, we found nuanced associations between sleep architecture and affect across dimensions of arousal and valence. Extension of SWS and REM sleep, while considering trade-offs in other stages (light sleep and awake in bed), predicted better post-sleep morning affect. Findings from this study highlight the importance of considering sleep architecture as an integrated composition and using Bayesian multilevel compositional data analysis to enhance experimental and longitudinal studies and our understanding of the multifaceted association between sleep and affect.

## Supplementary Material

kaaf050_suppl_Supplementary_Material

## Teaser

A novel, Bayesian multilevel compositional data analysis revealed that people with more deep sleep and rapid eye movement sleep, offset by light sleep or time awake in bed, have better emotional well-being in everyday life.

## Transparency Statement

Study materials are available at https://doi.org/10.17605/OSF.IO/TZ48Y. The data that support the findings of this study are available from the corresponding authors upon request. The analysis was not pre-registered. All analytical code is available at https://github.com/florale/multilevelcoda-sleep-stages-affect.
