# Correction to: Daily, prospective associations between sleep architecture and affect: insights from Bayesian multilevel compositional data analysis

**DOI:** 10.1093/abm/kaaf089

**Published:** 2025-12-08

This is a correction to: Flora Le, Yang Yap, Dorothea Dumuid, Joshua F Wiley, Daily, prospective associations between sleep architecture and affect: insights from Bayesian multilevel compositional data analysis, *Annals of Behavioral Medicine*, Volume 59, Issue 1, 2025, https://doi.org/10.1093/abm/kaaf050

In the originally published version of the manuscript the legends to two figures were errored. The legend to Figure 3 should read: “[…] For example, the top panel shows reallocation between SWS and other sleep stages, where positive values on the x-axis (e.g., +60 minutes) indicate reallocations from another stage to SWS, whereas negative values (e.g., –60 minutes) indicate reallocations from SWS to another stage.” instead of: “[…] For example, the top panel shows reallocation between SWS and other sleep stages, where positive values on the x-axis (e.g., +30 minute) indicate reallocations of from SWS to another stage, whereas negative values (e.g., –30) indicate reallocations of from another stage to SWS.”.

The legend to Figure 4 should read: “[…] For example, the top panel shows reallocation between SWS and other sleep stages, where positive values on the x-axis (e.g., +60 minutes) indicate reallocations from another stage to SWS, whereas negative values (e.g., –60 minutes) indicate reallocations from SWS to another stage.” instead of: “[…] For example, the top panel shows reallocation between SWS and other sleep stages, where positive values on the x-axis (e.g., +30 minute) indicate reallocations of from SWS to another stage, whereas negative values (e.g., –30) indicate reallocations of from another stage to SWS.”.

These emendations have been made in the article.

