# Supplementary material for: Daily, prospective associations between sleep architecture and affect: insights from Bayesian multilevel compositional data analysis
Source: Ann Behav Med. 2025 Jun 25;59(1):kaaf050. doi: 10.1093/abm/kaaf050 (PMC12208346; doi:10.1093/abm/kaaf050)
Supplement: kaaf050_suppl_Supplementary_Material [file kaaf050_suppl_supplementary_material.html]

Supplementary materials: Daily, prospective associations between sleep architecture and affect: insights from Bayesian multilevel compositional data analysis


# Supplementary materials: Daily, prospective associations between sleep architecture and affect: insights from Bayesian multilevel compositional data analysis

#### Flora Le (flora.le@monash.edu)

- 1 Scatterplots of raw sleep
  architecture and affect data
- 2 Statistics for 30-minute
  reallocations
- 3 Figures for reallocations from 1 to
  60 minutes
  - 3.1 Between person
  - 3.2 Within person

This file presents the full daily reallocation of sleep stages and
affect results using the Bayesian compositional multilevel substitution
analysis for the manuscript: **Daily, Prospective Associations
between Sleep Architecture and Affect: Insights from Bayesian Multilevel
Compositional Data Analysis**.

# 1 Scatterplots of raw sleep architecture and affect data

*Notes*. The plots below show the bivariate associations
between sleep architecture and affect, without accounting for the
compositional nature of the data. **High arousal positive
affect**

**Low arousal positive affect**

**High arousal negative affect**

**Low arousal negative affect**

# 2 Statistics for 30-minute reallocations

*Notes*. All estimates are relative to the mean sleep
architecture composition. TWT = total wake time in bed, Light = light
sleep, SWS = slow wave sleep, REM = rapid eye movement sleep. \*
indicates 95% credible intervals not containing 0. Models adjusted for
age, sex, race/ethnicity, subjective social status, body mass index,
weekdays/weekend, circadian misalignment, and previous-night pre-sleep
affect.

**High arousal positive affect**

| Estimate [95% CI] | Minute | From | To | Level | Sig |
| --- | --- | --- | --- | --- | --- |
| -0.48 [-0.89, -0.07] | 30 | Slow Wave Sleep | Light Sleep | between | Yes |
| 0.10 [-0.31, 0.50] | 30 | REM Sleep | Light Sleep | between |  |
| -0.06 [-0.44, 0.34] | 30 | Total Wake Time | Light Sleep | between |  |
| 0.38 [ 0.04, 0.72] | 30 | Light Sleep | Slow Wave Sleep | between | Yes |
| 0.46 [ 0.02, 0.91] | 30 | REM Sleep | Slow Wave Sleep | between | Yes |
| 0.31 [-0.14, 0.76] | 30 | Total Wake Time | Slow Wave Sleep | between |  |
| -0.04 [-0.39, 0.31] | 30 | Light Sleep | REM Sleep | between |  |
| -0.53 [-1.00, -0.07] | 30 | Slow Wave Sleep | REM Sleep | between | Yes |
| -0.11 [-0.43, 0.21] | 30 | Total Wake Time | REM Sleep | between |  |
| 0.08 [-0.24, 0.39] | 30 | Light Sleep | Total Wake Time | between |  |
| -0.42 [-0.87, 0.03] | 30 | Slow Wave Sleep | Total Wake Time | between |  |
| 0.16 [-0.14, 0.47] | 30 | REM Sleep | Total Wake Time | between |  |
| 0.00 [-0.08, 0.08] | 30 | Slow Wave Sleep | Light Sleep | within |  |
| -0.05 [-0.14, 0.05] | 30 | REM Sleep | Light Sleep | within |  |
| 0.00 [-0.11, 0.10] | 30 | Total Wake Time | Light Sleep | within |  |
| 0.00 [-0.06, 0.07] | 30 | Light Sleep | Slow Wave Sleep | within |  |
| -0.05 [-0.14, 0.05] | 30 | REM Sleep | Slow Wave Sleep | within |  |
| 0.00 [-0.09, 0.09] | 30 | Total Wake Time | Slow Wave Sleep | within |  |
| 0.04 [-0.04, 0.12] | 30 | Light Sleep | REM Sleep | within |  |
| 0.04 [-0.05, 0.13] | 30 | Slow Wave Sleep | REM Sleep | within |  |
| 0.03 [-0.07, 0.13] | 30 | Total Wake Time | REM Sleep | within |  |
| 0.01 [-0.08, 0.09] | 30 | Light Sleep | Total Wake Time | within |  |
| 0.01 [-0.07, 0.08] | 30 | Slow Wave Sleep | Total Wake Time | within |  |
| -0.04 [-0.13, 0.05] | 30 | REM Sleep | Total Wake Time | within |  |

**Low arousal positive affect**

| Estimate [95% CI] | Minute | From | To | Level | Sig |
| --- | --- | --- | --- | --- | --- |
| -0.48 [-0.89, -0.07] | 30 | Slow Wave Sleep | Light Sleep | between | Yes |
| 0.10 [-0.31, 0.50] | 30 | REM Sleep | Light Sleep | between |  |
| -0.06 [-0.44, 0.34] | 30 | Total Wake Time | Light Sleep | between |  |
| 0.38 [ 0.04, 0.72] | 30 | Light Sleep | Slow Wave Sleep | between | Yes |
| 0.46 [ 0.02, 0.91] | 30 | REM Sleep | Slow Wave Sleep | between | Yes |
| 0.31 [-0.14, 0.76] | 30 | Total Wake Time | Slow Wave Sleep | between |  |
| -0.04 [-0.39, 0.31] | 30 | Light Sleep | REM Sleep | between |  |
| -0.53 [-1.00, -0.07] | 30 | Slow Wave Sleep | REM Sleep | between | Yes |
| -0.11 [-0.43, 0.21] | 30 | Total Wake Time | REM Sleep | between |  |
| 0.08 [-0.24, 0.39] | 30 | Light Sleep | Total Wake Time | between |  |
| -0.42 [-0.87, 0.03] | 30 | Slow Wave Sleep | Total Wake Time | between |  |
| 0.16 [-0.14, 0.47] | 30 | REM Sleep | Total Wake Time | between |  |
| 0.00 [-0.08, 0.08] | 30 | Slow Wave Sleep | Light Sleep | within |  |
| -0.05 [-0.14, 0.05] | 30 | REM Sleep | Light Sleep | within |  |
| 0.00 [-0.11, 0.10] | 30 | Total Wake Time | Light Sleep | within |  |
| 0.00 [-0.06, 0.07] | 30 | Light Sleep | Slow Wave Sleep | within |  |
| -0.05 [-0.14, 0.05] | 30 | REM Sleep | Slow Wave Sleep | within |  |
| 0.00 [-0.09, 0.09] | 30 | Total Wake Time | Slow Wave Sleep | within |  |
| 0.04 [-0.04, 0.12] | 30 | Light Sleep | REM Sleep | within |  |
| 0.04 [-0.05, 0.13] | 30 | Slow Wave Sleep | REM Sleep | within |  |
| 0.03 [-0.07, 0.13] | 30 | Total Wake Time | REM Sleep | within |  |
| 0.01 [-0.08, 0.09] | 30 | Light Sleep | Total Wake Time | within |  |
| 0.01 [-0.07, 0.08] | 30 | Slow Wave Sleep | Total Wake Time | within |  |
| -0.04 [-0.13, 0.05] | 30 | REM Sleep | Total Wake Time | within |  |

**High arousal negative affect**

| Estimate [95% CI] | Minute | From | To | Level | Sig |
| --- | --- | --- | --- | --- | --- |
| 0.21 [ 0.00, 0.42] | 30 | Slow Wave Sleep | Light Sleep | between |  |
| -0.04 [-0.24, 0.17] | 30 | REM Sleep | Light Sleep | between |  |
| -0.10 [-0.29, 0.09] | 30 | Total Wake Time | Light Sleep | between |  |
| -0.16 [-0.33, 0.02] | 30 | Light Sleep | Slow Wave Sleep | between |  |
| -0.19 [-0.42, 0.04] | 30 | REM Sleep | Slow Wave Sleep | between |  |
| -0.26 [-0.49, -0.03] | 30 | Total Wake Time | Slow Wave Sleep | between | Yes |
| 0.02 [-0.16, 0.20] | 30 | Light Sleep | REM Sleep | between |  |
| 0.23 [-0.01, 0.48] | 30 | Slow Wave Sleep | REM Sleep | between |  |
| -0.08 [-0.24, 0.09] | 30 | Total Wake Time | REM Sleep | between |  |
| 0.05 [-0.10, 0.21] | 30 | Light Sleep | Total Wake Time | between |  |
| 0.27 [ 0.04, 0.50] | 30 | Slow Wave Sleep | Total Wake Time | between | Yes |
| 0.02 [-0.14, 0.18] | 30 | REM Sleep | Total Wake Time | between |  |
| -0.01 [-0.05, 0.03] | 30 | Slow Wave Sleep | Light Sleep | within |  |
| 0.05 [ 0.00, 0.10] | 30 | REM Sleep | Light Sleep | within |  |
| -0.01 [-0.06, 0.04] | 30 | Total Wake Time | Light Sleep | within |  |
| 0.01 [-0.03, 0.04] | 30 | Light Sleep | Slow Wave Sleep | within |  |
| 0.06 [ 0.00, 0.11] | 30 | REM Sleep | Slow Wave Sleep | within |  |
| -0.01 [-0.05, 0.04] | 30 | Total Wake Time | Slow Wave Sleep | within |  |
| -0.04 [-0.08, 0.00] | 30 | Light Sleep | REM Sleep | within |  |
| -0.05 [-0.10, 0.00] | 30 | Slow Wave Sleep | REM Sleep | within |  |
| -0.05 [-0.10, 0.00] | 30 | Total Wake Time | REM Sleep | within |  |
| 0.00 [-0.03, 0.04] | 30 | Light Sleep | Total Wake Time | within |  |
| -0.01 [-0.05, 0.03] | 30 | Slow Wave Sleep | Total Wake Time | within |  |
| 0.05 [ 0.01, 0.10] | 30 | REM Sleep | Total Wake Time | within | Yes |

**Low arousal negative affect**

| Estimate [95% CI] | Minute | From | To | Level | Sig |
| --- | --- | --- | --- | --- | --- |
| 0.09 [-0.14, 0.32] | 30 | Slow Wave Sleep | Light Sleep | between |  |
| -0.18 [-0.40, 0.04] | 30 | REM Sleep | Light Sleep | between |  |
| -0.18 [-0.40, 0.04] | 30 | Total Wake Time | Light Sleep | between |  |
| -0.05 [-0.23, 0.14] | 30 | Light Sleep | Slow Wave Sleep | between |  |
| -0.23 [-0.48, 0.02] | 30 | REM Sleep | Slow Wave Sleep | between |  |
| -0.23 [-0.49, 0.03] | 30 | Total Wake Time | Slow Wave Sleep | between |  |
| 0.15 [-0.04, 0.34] | 30 | Light Sleep | REM Sleep | between |  |
| 0.24 [-0.03, 0.50] | 30 | Slow Wave Sleep | REM Sleep | between |  |
| -0.04 [-0.22, 0.15] | 30 | Total Wake Time | REM Sleep | between |  |
| 0.13 [-0.04, 0.30] | 30 | Light Sleep | Total Wake Time | between |  |
| 0.22 [-0.03, 0.48] | 30 | Slow Wave Sleep | Total Wake Time | between |  |
| -0.05 [-0.23, 0.12] | 30 | REM Sleep | Total Wake Time | between |  |
| 0.03 [-0.01, 0.07] | 30 | Slow Wave Sleep | Light Sleep | within |  |
| 0.02 [-0.03, 0.07] | 30 | REM Sleep | Light Sleep | within |  |
| -0.06 [-0.11, 0.00] | 30 | Total Wake Time | Light Sleep | within |  |
| -0.02 [-0.06, 0.01] | 30 | Light Sleep | Slow Wave Sleep | within |  |
| 0.00 [-0.06, 0.05] | 30 | REM Sleep | Slow Wave Sleep | within |  |
| -0.08 [-0.13, -0.03] | 30 | Total Wake Time | Slow Wave Sleep | within | Yes |
| -0.01 [-0.06, 0.03] | 30 | Light Sleep | REM Sleep | within |  |
| 0.02 [-0.03, 0.07] | 30 | Slow Wave Sleep | REM Sleep | within |  |
| -0.07 [-0.12, -0.02] | 30 | Total Wake Time | REM Sleep | within | Yes |
| 0.03 [-0.01, 0.08] | 30 | Light Sleep | Total Wake Time | within |  |
| 0.06 [ 0.02, 0.11] | 30 | Slow Wave Sleep | Total Wake Time | within | Yes |
| 0.05 [ 0.00, 0.10] | 30 | REM Sleep | Total Wake Time | within |  |

# 3 Figures for reallocations from 1 to 60 minutes

*Notes*. All estimates are relative to the mean sleep
architecture composition. TWT = total wake time in bed, Light = light
sleep, SWS = slow wave sleep, REM = rapid eye movement sleep. \*
indicates 95% credible intervals not containing 0. Models adjusted for
age, sex, race/ethnicity, subjective social status, body mass index,
weekdays/weekend, circadian misalignment, and previous-night pre-sleep
affect.

## 3.1 Between person

## 3.2 Within person
